# Modulation of Gene Expression in Contextual Fear Conditioning in the Rat

**DOI:** 10.1371/journal.pone.0080037

**Published:** 2013-11-21

**Authors:** Giuseppe Federighi, Giovanna Traina, Monica Macchi, Cristina Ciampini, Rodolfo Bernardi, Elisabetta Baldi, Corrado Bucherelli, Marcello Brunelli, Rossana Scuri

**Affiliations:** 1 Dipartimento di Ricerca Traslazionale e Delle Nuove Tecnologie in Medicina e Chirurgia, Unità di Fisiologia, Università di Pisa, Pisa, Italy; 2 Dipartimento di Scienze Economico-Estimative e degli Alimenti, Sezione di Chimica Bromatologica, Biochimica, Fisiologia e Nutrizione, Università degli Studi di Perugia, Perugia, Italy; 3 Dipartimento di Biologia, Università di Pisa, Pisa, Italy; 4 Dipartimento di Scienze Agrarie, Genetica Alimentari e Agro-Ambientali, Università di Pisa, Pisa, Italy; 5 Dipartimento di Scienze Fisiologiche, Università di Firenze, Firenze, Italy; Harbin Institute of Technology, China

## Abstract

In contextual fear conditioning (CFC) a single training leads to long-term memory of context-aversive electrical foot-shocks association. Mid-temporal regions of the brain of trained and naive rats were obtained 2 days after conditioning and screened by two-directional suppression subtractive hybridization. A pool of differentially expressed genes was identified and some of them were randomly selected and confirmed with qRT-PCR assay. These transcripts showed high homology for rat gene sequences coding for proteins involved in different cellular processes. The expression of the selected transcripts was also tested in rats which had freely explored the experimental apparatus (exploration) and in rats to which the same number of aversive shocks had been administered in the same apparatus, but temporally compressed so as to make the association between painful stimuli and the apparatus difficult (shock-only). Some genes resulted differentially expressed only in the rats subjected to CFC, others only in exploration or shock-only rats, whereas the gene coding for translocase of outer mitochondrial membrane 20 protein and nardilysin were differentially expressed in both CFC and exploration rats. For example, the expression of stathmin 1 whose transcripts resulted up regulated was also tested to evaluate the transduction and protein localization after conditioning.

## Introduction

Memory is the capacity to retain learned information. On the basis of its duration, we can distinguish short-term (ST) and long-term (LT) memories which are accompanied by different biological states and mechanisms of retention and therefore they are two distinct forms of memory. In ST memory, transient and non-stabilized post-translational modifications of pre-existing molecules [1] allow the retention of information for minutes or hours without creating persistent neural changes for later recall [2], [3]. LT memory occurs because changes in neural pathways take place for the storage of information that can be recalled weeks, months, or years later. Studies in both *Aplysia* and *Drosophila* systems allowed the discovery of the first molecular pathway essential for memory consolidation, that is the cyclic AMP (cAMP)-protein kinase A (PKA)-cAMP response element-binding protein (CREB)-CCAAT enhancer-binding protein (C/EBP)-dependent pathway [4], [5]. Subsequently, increasing evidence has shown, in several learning tasks and different species, that induction of LT memory depends on the temporally limited phase of mRNA and protein synthesis so that transcription, like translation, is an essential step for memory formation [6]–[9] and memory consolidation requires de novo mRNA and protein synthesis for several hours [8], [10]–[12], [13]–[15]. Initially these studies enjoyed support by using inhibitors of mRNA transcription in different behavioural paradigms and in various animal models ranging from sea snails and goldfish to rats [16]–[19]. More recently the use of technologies to quantify alterations in gene expression in brain tissue has provided new and important insights about how gene expression is altered by experience and how these molecular changes may provide a substrate for the LT storage of new memories.

Pavlovian fear conditioning in rodents is one of the most productive approaches to understand the neurobiology of learning and memory. This robust form of learning is rapidly acquired, straightforward to measure, and relies on a fairly well-described neural circuit that includes cortical and subcortical areas. In the contextual fear conditioning (CFC) paradigm, an emotionally neutral conditioned stimulus (CS, e.g., contextual stimulus) is paired with an aversive unconditioned stimulus, (US, e.g., a foot shock), during the acquisition phase. As a result, the CS comes to elicit a conditioned fear response during the expression phase, without the need for foot shocks, because it acquires aversive reinforcing properties [20], [21].

Rats subjected to CFC procedure exhibit long-lasting freezing (complete absence of somatic motility, respiratory movements excepted) when placed in the conditioning apparatus again, whereas they do not exhibit freezing when placed in surroundings different from those in which the aversive stimuli have been administered [22], [23]. Therefore, the freezing response appears to be a specific conditioned response due to the association of specific surroundings and the aversive stimuli. In a previous study [24] it has been showed that CFC rats exhibited the freezing response for no less than 4 weeks and the hippocampal excitability measured in brain slices was increased for 7 days.

Because CFC induces long lasting behavioural and cellular changes, in the present study we have chosen to investigate the gene expression 2 days after conditioning during the consolidation and retention of the freezing response in order to clarify the mechanisms underlying memory storage at the genetic level.

## Materials and Methods

### Ethics Statement

All experiments were carried out in strict accordance with the recommendations in the Guide for the Care and Use of Laboratory Animals published by the US National Institutes of Health (NIH publication No. 85–23). The protocol was approved by the Committee on the Ethics of Animal Experiments of the University of Florence (Prot. N. 77/2008).

### Animals

Normal male Wistar rats older than 70 days (Charles River, Calco, Italy), were employed. 25 rats were subjected to contextual fear conditioning (CFC) and subsequently tested behaviorally or biologically (SSH analysis, qRT-PCR, Western Blot and immunofluorescence analysis) at 2 days post-acquisition; 10 rats were subjected to a shock-only procedure (see below) and 10 rats were subjected to an exploration procedure (see below) and all afterward tested behaviorally or sacrificed at 2 days to perform qRT-PCR. Groups of 5 rats (naïve) which had never left the home cage were employed as controls.

Rats belonged to the same brood in order to avoid genetic variability in the groups used.

The animals were kept in a room under natural daylight rhythm at a constant temperature of 20±1°C. The rats had access to food and water *ad libitum* throughout the experiment.

To obtain sample of mid-temporal regions including hippocampus and amygdala, animals were anesthetized with ether and then killed by decapitation. Brains were quickly removed, dissected, frozen and stored at −80°C until use.

Both behavioural and biological measurements were performed by operators blind to the group from which rats or brain were taken.

### Behavioural procedures

Basic Skinner box module (dimensions 29×31×26 cm; Modular Operant Cage, Coulbourn Instruments L.L.C., Allentown, PA, USA) was used to induce freezing. The top and two opposite sides consisted of aluminium panels, the other two sides of transparent plastic. The floor was made of stainless steel rods connected to a shock scrambler delivery apparatus (Grid Floor Shocker, model E13-08, Coulbourn). The number and duration of the electric shocks and the duration of the intervals between them were predetermined with a stimulus programming device (Scatola di comando Arco 2340, Ugo Basile, Comerio, Varese, Italy) connected to the apparatus. The conditioning apparatus was placed in an acoustically insulated room kept at a constant temperature of 20±1°C. Illumination inside the room was 60 lux.

### Contextual fear conditioning procedure

The rat was gently taken manually from the home cage, placed in a bucket and carried from the housing room to the soundproofed room. Once there, it was placed inside the conditioning apparatus where it was left undisturbed for 3 min. Afterward, a series of seven single electric foot-shocks (1 sec duration, 1 mA intensity) were administered at 30 sec intervals. Two minutes after the end of the stimulation, the rats were brought back to the home cage, thus spending altogether 8 min inside the conditioning apparatus.

### Shock-only procedure

As in CFC procedure, the rat was gently taken manually from the home cage, placed in a bucket and carried from the housing room to the soundproofed room. Once there, it was placed inside the conditioning apparatus. Immediately afterwards the rat was subjected to seven foot-shocks (1 sec duration, 1 mA intensity) at 3 sec intervals. At the end of the stimulation, the rat was brought back to the home cage, thus spending a total time of about 30 sec inside the conditioning apparatus.

### Exploration procedure

As in CFC procedure, the rat was gently taken manually from the home cage, placed in a bucket and carried from the housing room to soundproofed room. Once there, it was placed inside the conditioning apparatus where it was left undisturbed for 8 min before being brought back to the home cage.

### Freezing measurement

To measure freezing, the rats were again placed inside the conditioning apparatus and left there for 3 min. Afterward, they were brought back to the home cage. The behaviour of the rats was recorded by means of a closed-circuit television system. The total amount of freezing (in seconds) during the 3 min period (total freezing) was measured manually with a stop-watch. All behavioural testing was performed between 10.00 and 13.00 h to minimize circadian influences.

### Suppression subtraction hybridization (SSH) protocol

Suppression subtractive hybridization (SSH) was performed in accordance with the protocol previously described by Traina et al. [25]. Briefly, total RNA was isolated from brain samples of 5 rats sacrificed 2 days after CFC and 5 naive rats. 2 mg of poly (A)^+^ RNAs were purified from the pools of total RNAs using the PolyATtract mRNA Isolation System (Promega Corp., Madison, WI). PCR-select cDNA subtraction was performed using PCR-Select™ cDNA Subtraction Kit (Diatech Lab Line Srl, Jesi, Italy) according to the manufacturer's protocol. cDNAs from brain tissue of rats subjected to CFC were used as *tester* and cDNAs from naive rats as *driver* in forward subtraction and *vice versa* in reverse subtraction. The purified secondary SSH-PCR products were cloned into PCR® II-TOPO vector (TOPO TA Cloning® kit, Life Technologies Italia, Milan, Italy). The positive clones were collected and stored after growing in microtetra plates. A differential screening was performed for all collected clones using the cDNA Array approach (Clontech PCR-Select Differential Screening kit, Diatech Lab Line Srl, Jesi, Italy). Few microliters of the stored positive colonies in freezing broth were inoculated into 10 ml of LB-ampicillin (100 mg L^−1^) and were grown overnight at 37°C. Plasmid DNAs were prepared using Wizard PLUS SV Minipreps DNA Purification System Promega Corp., Madison, WI) according to the manufacturer's protocol and sequenced by automated sequencing (MWG Biotech, Ebersberg, Germany). cDNA sequences were submitted to database search *GeneBank* by FASTA: (http://www.ebi.ac.uk/fasta33/) and BLAST: (http://www.ch.embnet.org/software/aBLAST.html).

### qRT-PCR

Total RNA was extracted in ClCs gradient and single strand cDNAs were synthesized using iScript Select (Bio-Rad, Milan, Italy) kit. Quantitative real-time PCR was performed with SYBR Green kit on the MiniOpticon Two-Color Real-time PCR detection system (Bio-Rad, Milan, Italy). The primers used were constructed using the software Beacon Designer 7 (Bio-Rad, Milan, Italy). The forward and reverse primers were chosen to hybridize a single specific region of the appropriate gene sequence and are listed in Table 1.

**Table 1:** Primer sequences used in q-RT-PCRs assay.

Gene	Primer sequences	Accession N°
N-ethylmaleimide-sensitive factor attachment protein, alpha (Napa)	F:CAGTCCCCTCCTCAAGTACAGC R:TCCTCATACTTCTGAACAGCAAGC	NM_080585.1
profilin-2 (Pfn2)	F:GCAGCCTCTCCCATCTACCT R:ACCCTTGGGAATCATGACAG	NM_030873.1
caspase 3 (Casp3)	F:GCTGTCAGTCAGAGCGTAAG R:CACAAGGTGGGTCCAACTAGC	NM_012922.2
p53 and DNA damage regulated 1 (Pdrg1)	F:CTCTCAGACAAGCGGCAGATTG R:TGTTCCCAAAGCAAACCATCACG	NM_001014762.1
tyrosine 3-monooxygenase/tryptophan 5-monooxygenase activation protein, zeta polypeptide (Ywhaz)	F:AGGCTTGGAGCACTTGTGAG R:GTGAACCGTTTCTGCCCTTA	NM_013011.3
stathmin 1 (Stmn1)	F:GTGCTGTATTGACTGTGGAAGAC R:AGAATTGGGATCGCAAAGTGAAC	NM_017166.1
2,3-bisphosphoglicerate mutase (Bpgm)	F: ACGACCAGGAGCAAGCAGAG R:AAATCAAACGGGAGGAGCAGAAG	NM_199382.1
actin related protein 3 homolog (Actr3)	F:CATTGTCCTCTCTGGTGGTTCAAC R:CTCAACTTCAGCCTGGCATCTAC	NM_031068.1
phosphoprotein enriched in astrocytes 15 (Pea15)	F:CAGGCAGGACTAGGGAGACTT R:AAACATTGCATTTACATTTGAGC	NM_001013231.1
TIP41, TOR signaling pathway regulator-like (Tiprl)	F:CAGAGCAGCCACCAGGACTTC R:AGGGCATCCGTAGCATTGAACTC	NM_001109667.1
Complexin 1 (Cplx1)	F:AAGGGACTCCAAGTGTGTCG R:GAAATGAGCAGTGGGTGCTT	NM_022864.3
tripartite motif-containing 32 (Trim32)	F: CGAAGCCCACATAATCCAAT R:CACAAAGTCCCTTTGGGAAG	NM_001012103.1
RAN, member RAS oncogene family (Ran)	F: AGCCGCAGGTCCAGTTCAAG R: TGATGGGTCCTCTGTTGGTATG	NM_053439.1
amphyphisin-like (Amph2)	F:GACCCACTCACCCATGTCTC R:AAATCAGTGCTTGTCGTCCA	XM_003751705.1
translocase of outer mitochondrial membrane 20 homolog (Tomm20)	F:TACAACAGACTCTTCCACCACCAG R:CATTCCACATCATCCTCAGCCAAG	NM_152935.1
nardilysin 1 (Nrd1)	F:GAGCGGATGAATCTGAGGAGGAG R:TTTGATGTATCGGTACTGCTTGGG	NM_012993.2
G3PDH	F:CGACCTTCACCATCTTGCCTATGA R:GCTCTCTGCTCCTCCCTGTTC	NM_017008.4

For the PCR reaction 15 µL mastermix were prepared using 7.5 µL iQ Sybr Green Supermix (200 µM dNTP, 5 mM MgCl2, 3.75 U iTaqTM DNA polymerase), 1 µg/µl cDNA, 300 nM of each primer and RNase-free H2O. Cycling conditions included initial denaturation (3 min at 95°C), amplification, and quantification program repeated 40 times (10 sec at 95°C, 60 sec at 59°C with a single fluorescent measurement at the end of each elongation step) and dissociation protocol (60°C to 95°C by 1°C increments followed by a 30 sec hold and fluorescent measurement).

According to the protocol indicated by Bio-Rad (Milan, Italy), primers' efficiency has been previously assessed. Each target gene was loaded with the glyceraldehyde 3-phosphate dehydrogenase (G3PDH), used as housekeeping gene.

Before using G3PDH we tested three different candidate housekeeping genes: G3PDH, actin and RPL13A. In three biological replicates for each of which we done three technical replicates, we evaluated the C_t_ value in samples from naïve, conditioned (CFC), shock-only (SO) and exploration (explor) rats and the C_t_ value of both G3PDH and RPL13A were comparable with each of the genes we analyzed in this work and showed small changes in the various samples. Therefore, G3PDH and RPL13A were the best housekeeping genes and we have chosen to utilize the G3PDH because we had used it in previous experiments. Instead, the actin gene had a high number of cycles and the C_t_ value in the various samples showed variations (see Fig. S1 and Tab. S1).

Changes in the expression were assessed by the method of 2-ΔΔC_T_ [26]: the expression levels of each target gene were normalized to the G3PDH gene expression level. This method assumes that the gene is amplified with an efficiency close to 100% and not less than 5%.

### Western Blotting

Two days after training, 5 rats subjected to CFC and 5 naïve rats were sacrificed to obtain samples of brain mid-temporal areas. Tissue was then lysed (lysis buffer:1 mM EDTA, TRITON x-100 0,5%, 5 mM NaF, 6 M Urea, 1 mM Na- 43 orthovanadate activated, 2,5 mM Na-pyrophosphate), samples loaded, and proteins separated by SDS-PAGE according to Traina et al. [27]. Proteins were then transferred to nitrocellulose membrane and immunoblotted with primary antibodies recognizing stathmin 1 (Santa Cruz Biotechnology Inc., CA, USA) and subsequently incubated with secondary antibodies goat anti-mouse IgG-HRP (H&L) (Chemicon International, Inc. Pittsburg, PA, USA). Blots were detected with Lumi-Glo ECL substrate (Kit Super Signal West Pico Chemioluminescent Substrat, Rockford, IL, USA) and captured with UVP Image Store 5000 chemiluminescent imaging system.

The relative expression of stathmin 1 was determined by normalizing the integrated band density values to the values obtained for G3PDH on the same blots (Quantity One® Software, Bio-Rad, Milan, Itlay).

### Immunofluorescence analysis

In order to carry out the immunofluorescence analysis on hippocampal slices 2 days after CFC, 5 rats subjected to CFC and 5 naïve rats were previously perfused intracardially with a standard p-formaldehyde fixative solution. Briefly, the animals were anesthetized (80 mg/kg of sodium pentobarbital intraperitoneally) and the thoracic cavity was opened and a (polished) 21-gauge needle connected to the gravity-dependent perfusion system was inserted through the left ventricle into the ascending aorta. Then, an incision was made in the right atrium to drain incoming venous blood and the perfusion solution was allowed to enter into the left ventricle. Bottles of the perfusion system were placed 120 cm above the animal (10 mL/min). The perfusion was performed with 500 mL of 4% p-formaldehyde (PFA) in 0.1 M phosphate buffer (PB, pH 7.2) solution. Brains were then dissected, post-fixed in PFA in PB for 6 h and protected (preserved) by immersion in PB with 25% sucrose and 0.01% sodium azide at 4°C until the use

The brains were embedded in Killik cryostat-embedding compound (Bio-Optica, Milan, Italy) cut into 20 µm thick sections on a cryostat (Leyca Microsystems,Germany) at −22 C, and mounted on slides. The brain sections were rinsed in 0.1 M PB and incubated for 48 h at 4°C in a 1∶500 rabbit polyclonal antibody (about 6 µg/mL) directed against the C-terminal of mammalian stathmin 1 (Calbiochem, EMD Chemicals, Inc. Gibbstown, USA). Triton X-100 at 0.3% was added diluted in 0.1 M PB. After incubation, the sections were rinsed in 0.1 M PB and incubated overnight at 4°C in the secondary antibody (Alexa Fluor-488; Molecular Probes, Eugene, OR, USA) at a dilution of 1: 200 in 0.1 M PB containing 0.3% Triton X-100. Finally, the sections were rinsed in 0.1 M PB, mounted on gelatin-coated glass slides, and cover slipped with a 0.1 M PB-glycerine mixture.

Control experiments were also performed to ensure that the secondary antibodies reacted only with the appropriate antigen–antibody complex. At this aim, staining controls were performed by incubating with PB instead of the primary antibody. Immunofluorescent materials were observed with a fluorescence microscopy system (Eclipse E800; Nikon, Badhoevedorp, The Netherlands) and images were acquired (DFC320 camera; Leica Microsystems, Wetzlar, Germany). Staining for AlexaFluor 488 was excited with a 488 nm wavelength and visualized as green fluorescence using 20× or 40× objective lens.

Electronic images from the microscope were processed using Adobe Photoshop 5.0 (Adobe Systems, Inc., Mountain View, CA, USA). Quantitative fluorescence data were exported from ImageJ (National Institute of Health, Bethesda, MD, USA) to calculate area and pixel value statistics and to generate density histograms.

### Statistical analysis

In the behavioural experiments, to detect significant differences among procedures one-way ANOVA and the Bonferroni multiple comparisons test were used.

The increase or decrease of gene expression at 2 days after conditioning was determined by comparing the target gene in CFC rats and control rats (naïve, exploration and shock-only) with a one way ANOVA design after normalization to G3PDH. All reactions were made in triplicate and data were plotted in histograms as mean ± E.S.

Unpaired *t* test was made to evaluate the significant differences in protein expression and optical density in immunofluorescence assay.

Statistics were carried out by using *GraphPad* Prism (version 4.0, Graph Pad Software Inc., San Diego, CA) and results were regarded as *not significant* if P>0.05.

## Results

### CFC induces a modulation of gene expression

**Figure 1:**
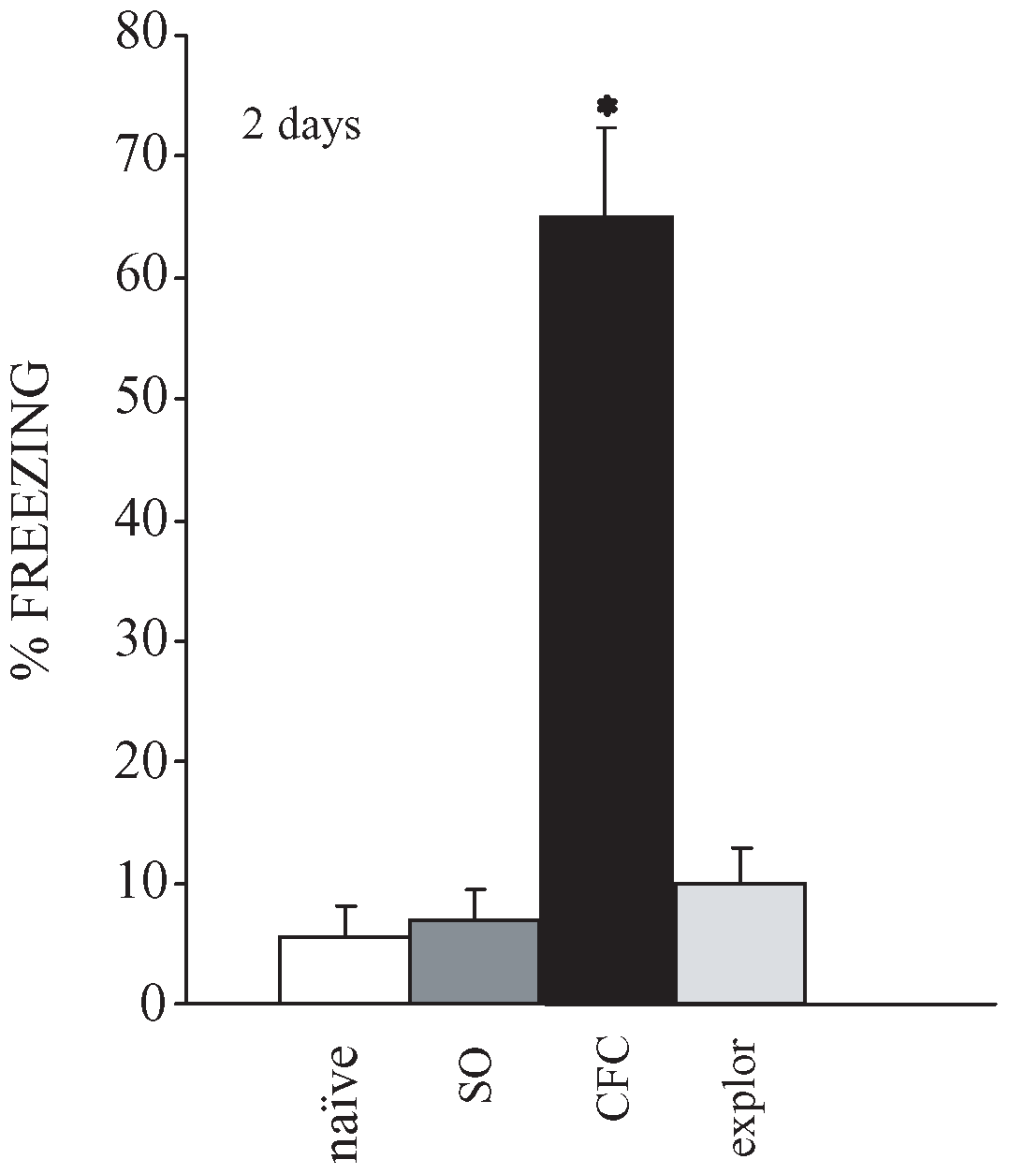
Freezing duration during retrieval testing performed 2 days after conditioning. For retrieval testing all rats were placed for 3 min in the apparatus and cumulated total freezing duration was measured. Freezing was defined as the complete absence of somatic motility, respiratory movements excepted. Conditioned rats (n = 5, CFC), which had received the electric foot-shocks during the 8 min period spent inside the apparatus exhibited freezing for about 65% of the time, whereas the rats never before placed in the apparatus (n = 5, naïve), the rats that had received 7 electric foot-shocks condensed in 30 sec (n = 5, SO) and the rats that had freely explored the apparatus for 8 min (n = 5, explor) exhibited a freezing duration < 10%. * indicates p < 0.001 among the 4 groups.

The aim of this research was to single out genes that are differentially expressed in the rat mid-temporal brain areas during the consolidation of CFC. For this reason, half of the animals used were behaviourally tested. All rats exhibited very similar behaviour when placed in the apparatus for the first time. As a rule, no initial freezing was observed, and only very short immobility durations (no more than 10% of total time) were recorded during the initial free-exploration 3 min period (data not shown). The rats which received, during the same training session, a series of 7 nociceptive shocks exhibited during the following 120 sec an almost continuous freezing, lasting about 80% of the total duration (data not shown). Figure 1 shows that conditioned rats (CFC) exhibited long-lasting freezing time when placed in the conditioning apparatus again at 2 days post-acquisition delays, while control rats (naïve) never before placed in the apparatus, “exploration” rats (explor) that had freely explored the apparatus and rats that had received the same number of electric foot-shocks, but temporally condensed (30 sec) (“shock only”, SO), exhibited a freezing duration <10%.

**Figure 2:**
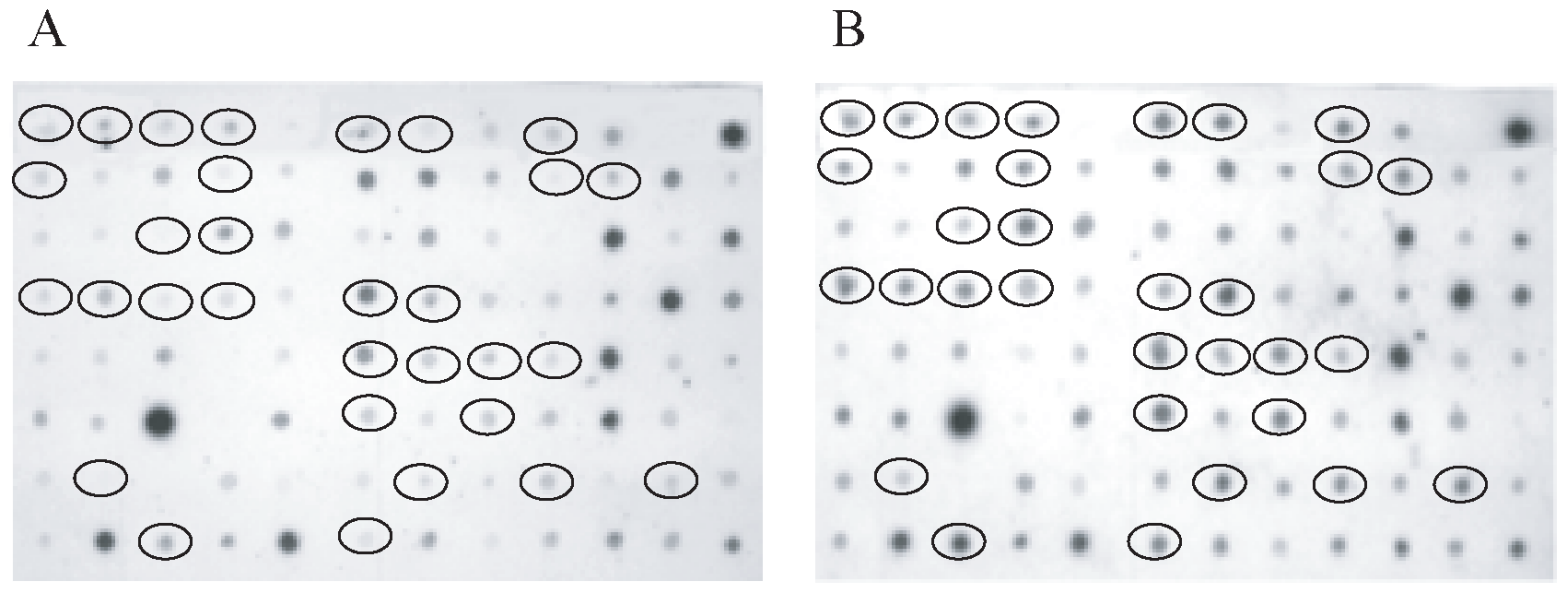
Differential screening of clones from forward library. Dot blot PCR products from forward subtractive clones were hybridized with cDNAs derived from the reverse library (A) and with cDNAs derived from the forward library (B). Circles indicate clones candidate to be differentially expressed in the CFC rats.

In order to detect modifications in gene expression related only to the consolidation and/or to the storage of engrams, if any, without the interference which may be consequent upon the repeated presentation of the stimuli received during acquisition, samples of rat mid-temporal brain areas were obtained at 2 days post-acquisition from 5 naïve rats and 5 rats subjected to CFC but not again to the stimuli of the training session. The total RNA was isolated to perform the suppression subtractive hybridization (SSH) method [25] for the construction of two subtractive cDNA libraries: the forward and reverse libraries consisting of transcripts positively and negatively modulated, respectively, by CFC. The screening of the positive clones was carried out by PCR-Select Differential Screening, using as primers the same nested primers used for the libraries amplification. Two identical blots were created by spotting PCR products and hybridised with cDNAs derived from forward- and reverse subtracted libraries. Figure 2 shows the differential screening of some clones from forward and reverse libraries.

About 1044 cDNA clones were collected for the forward cDNA library and 855 for the reverse cDNA library and the clones showing a single band after PCR amplification were sequenced.

**Figure 3:**
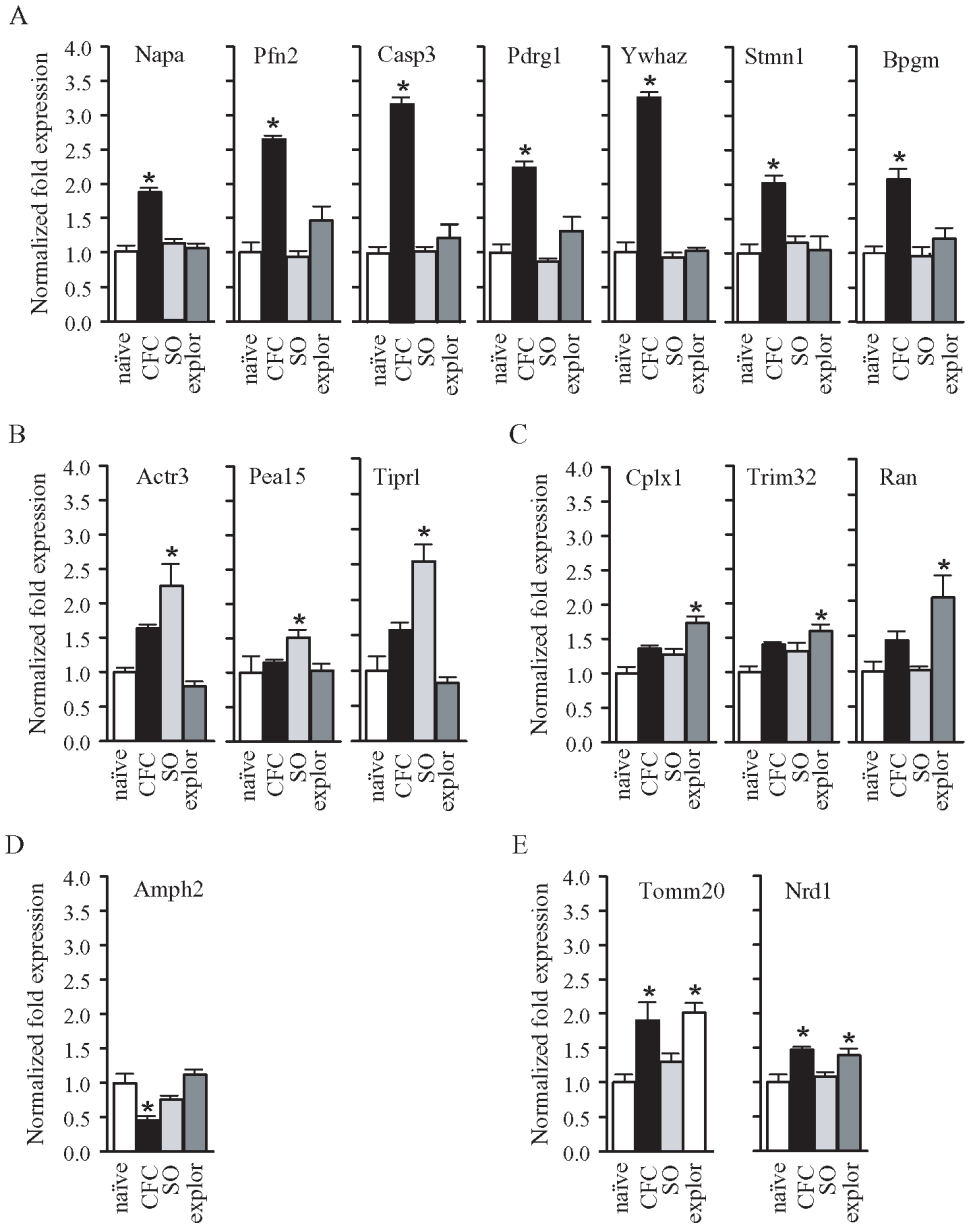
qRT PCR was carried out in brain samples obtained from naïve (n = 5), CFC (n = 5), SO (n = 5) and explor (n = 5) rats 2 days after conditioning to evaluate the expression levels of the selected genes. The transcript levels of each target gene were normalized to the G3PDH gene expression level and compared to the control sample (naïve). (A) The genes coding for Napa, Pfn2, Casp3, Pdrg1, Ywhaz, Stmn1 and Bpgm were significantly more expressed in CFC rats respect to all other groups of animals considered. (B) The genes coding for Actr3, Pea15 and Tiprl were significantly more expressed in SO animals and (C) the ones coding for Cplx1, Trim32 and Ran in exploration rats. (D) The gene coding for Amph2 was significantly less expressed in CFC rats in comparison with the other groups of animals considered and the genes coding for Tomm20 and Nrd1 respectively were significantly more expressed in both CFC and exploration rats with respect to naïve and SO rats.* indicates p<0.05.

We sequenced 178 differentially expressed cDNA clones, belonging to different cDNA libraries. With the information gathered from different databases we identified and assigned a putative function to 57 sequenced clones (Tables 2–3) believed to be interesting from a physiological point of view. We used qRT-PCR to analyze the expression of some of these clones randomly selected, not only in CFC and naïve rats but also in exploration and shock-only rats, in order to detect the gene whose expression was modulated by environment and aversive stimulus contextually. The results obtained were summarized in Table 4 and Figure 3 where the transcript levels of each target gene were normalized to the G3PDH gene expression level and compared to the control sample (naïve). Figure 3A shows that CFC positively modulates the expression of genes coding for: Napa (One Way ANOVA, F_3,25_ = 27,69, p = 0.0001; Bonferroni post-hoc test p<0.001), Pfn2 (One Way ANOVA, F_3,25_ = 22.25, p = 0.0001, Bonferroni post-hoc test p<0.001), Casp3 (One Way ANOVA, F_3,25_ = 44.72, p = 0.0001, Bonferroni post-hoc test p<0.001), Pdrg1 (One Way ANOVA, F_3,25_ = 15.12, p = 0.0001, Bonferroni post-hoc test p<0.001), Ywhaz (One Way ANOVA, F_3,25_ = 153.4, p = 0.0001, Bonferroni post-hoc test p<0.001), Stmn1 (One Way ANOVA, F_3,25_ = 8.23, p = 0.0007, Bonferroni post-hoc test p<0.01), Bpgm (One Way ANOVA, F_3,25_ = 10.58, p = 0.0002, Bonferroni post-hoc test p<0.001). Figure 3B shows that only the foot-shocks positively modulate the expression of genes coding for: Actr3 (One Way ANOVA, F_3,25_ = 11.50, p = 0.0001, Bonferroni post-hoc test p<0.05) Pea15 (One Way ANOVA, F_3,25_ = 3.54, p = 0.03, Bonferroni post-hoc test p<0.05), Tiprl (One Way ANOVA, F_3,25_ = 21.55, p = 0.0001, Bonferroni post-hoc test p<0.05). Figure 3C shows that the only context positively modulates the expression of genes coding for: Cplx1 (One Way ANOVA, F_3,25_ = 11.53, p = 0.0001, Bonferroni post-hoc test p<0.001), Trim32 (One Way ANOVA, F_3,25_ = 5.08, p = 0.008, Bonferroni post-hoc test p<0.01), Ran (One Way ANOVA, F_3,25_ = 6.19, p = 0.003, Bonferroni post-hoc test p<0.05). Figure 3D shows that CFC negatively modulates the expression of the gene coding for Amph2 (One Way ANOVA, F_3,25_ = 12.39, p = 0.0001, Bonferroni post-hoc test p<0.01). Finally, Figure 3E shows that CFC and context positively modulate the expression of genes coding for: Tomm20 (One Way ANOVA, F_3,25_ = 9.265, p = 0.0004, Bonferroni post-hoc test p<0.01 for both conditions) and Nrd1 (One Way ANOVA, F_3,25_ = 6.35, p = 0.003, Bonferroni post-hoc test p<0.05 for both conditions).

**Table 2:** Identification of up-regulated cDNA sequences.

Clone	Accession N° of matching sequence^(a)^	Putative identification^(b)^	Biological process^(c)^
1VA11	NM_001012103.1	tripartite motif-containing 32 (Trim32)	fat cell differentiation, negative regulation of apoptotic process and fibroblast proliferation, positive regulation of NF-kappaB transcription factor activity, cell cycle, cell growth, cell migration, neurogenesis, neuron differentiation, protein catabolic process, proteolysis, protein polyubiquitination, response to UV, response to tumor necrosis factor
1VA6	HM152028	ATP synthase F0 subunit 6 (Atp6)	ATP synthesis coupled proton transport
1VB12	NM_001100942.1	proteasome maturation protein (Pomp)	Ion transport, Transport
1VB4	NM_001013878.1	rattus norvegicus family with sequence similarity 149, member B1 (Fam149b1)	Unknown
1VB6	HM152028	cytochrome c oxidase subunit II (CoxII)	transport of electrons
1VB9	NM_001013231.1	phosphoprotein enriched in astrocytes 15 (Pea15)	Apoptosis, Sugar transport, Transport
1VC1	NM_021765.1	coatomer protein complex, subunit beta 2 (Copb2)	ER-Golgi transport, Protein transport, Transport
1VC10	NM_021880.2	protein kinase, cAMP dependent regulatory, type I, alpha (Prkar1a)	Signal transduction
1VD11	HM152028	cytochrome b (Cytb)	respiratory electron transport chain
1VE2	HM152028	cytochrome c oxidase subunit I (CoxI)	transport of electrons
1VE9	HM152028	NADH dehydrogenase subunit 6	NADH dehydrogenase (ubiquinone) activity
1VG5	NM_012993.2	nardilysin 1 (Nrd1)	Proteolysis
1VH5	NM_022864.3	complexin 1 (Cplx1)	Exocytosis, Neurotransmitter transport, Transport
2VB10	NM_031068.1	actin-related protein 3 homolog (Actr3)	Cilium biogenesis/degradation
2VB3	NM_001135819.1	pyridoxal (pyridoxine, vitamin B6) phosphatise (Pdxp)	Dephosphorylation
2VC5	NM_001170399.1	transmembrane protein 130 (Tmem130)	Unknown
2VC7	NM_080585.1	N-ethylmaleimide-sensitive factor attachment protein, alpha (Napa)	ER-Golgi transport, Protein transport, Transport
2VD11	NM_001126048.2	translational machinery associated 7 homolog(Tma7)	Unknown
2VE11	NM_001009622.1	SAR1 homolog B (Sar1b)	ER-Golgi transport, Protein transport, Transport
2VE2	XM_003751640.1	hypothetical protein	
2VE5	NM_00118719.2	tetratricopeptide repeat domain 7B (Ttc7b)	Unknown
2VE12	NM_030990.2	proteolipid protein 1 (Plp1)	Cell maturation, Glial cell differentiation, Integrin-mediated signalling pathway, Long-chain fatty acid biosynthetic process, Myelination, Positive regulation of gene expression
2VF11	NM_001024236.1	general transcription factor IIH, polypeptide 3 (Gtf2h3)	DNA damage, DNA repair, Transcription, Transcription regulation
2VG1	NM_013011.3	tyrosine 3-monooxygenase/tryptophan 5-monooxygenase activation protein, zeta polypeptide (Ywhaz)	Histamine secretion by mast cell, Protein targeting to mitochondrion, Regulation of cell death
2VG3	HM152028	NADH dehydrogenase subunit 6	NADH dehydrogenase (ubiquinone) activity
2VH1	NM_001106923.1	cullin 3 (Cul3)	ER-Golgi tran sport, Transport, Ubl conjugation pathway
3VD5	NM_001013184.1	high mobility group nucleosome binding domain 1 (Hmgn1)	Transcription, Transcription regulation
3VE1	NM_133568.1	RASD family, member 2 (Rasd2)	Apoptosis, DNA damage, Transcription, Transcription regulation
3VF10	NM_181626.3	iron-sulfur cluster assembly 1 homolog (Isca1)	Endocrine process, Iron-sulfur cluster assembly
3VG4	NM_009790.4	calmodulin 1 (Calm1)	regulation of cytokinesis
3VG5	NM_001004094.1	proteasome subunit alpha type 3-like (Psma3)	Ubiquitin-dependent protein catabolic process
3VH8	HM152028	cytochrome c oxidase subunit II (CoxII)	Transport of electrons
4VC11	NM_001024243.1	nudix (nucleoside diphosphate linked moiety X)-type motif 3(Nudt3)	Diphosphoinositol polyphosphate catabolic process
4VC4	NM_001109667.1	TIP41, TOR signaling pathway regulator-like (Tiprl)	DNA damage checkpoint, Negative regulation of protein phosphatase type 2A activity
4VE3	NM_024351.2	heat shock 70kDa protein 8 (Hspa8)	Stress response, Transcription, Transcription regulation, mRNA processing, mRNA splicing
4VF11	NM_001014174.1	similar to NICE-3 (LOC361985)	Unknown
4VF8	NM_152935.1	translocase of outer mitochondrial membrane 20 homolog (Tomm20)	Protein transport, Transport
4VH10	NM_001170601.1	signal recognition particle 72 (Srp54)	SRP-dependent cotranslational protein targeting to membrane, response to drug
5VD5	NM_030873.1	profilin 2 (Pfn2)	Actin cytoskeleton organization
5VD6	NM_012922.2	caspase 3 (Casp3)	Intracellular signal transduction/Apoptosis
5VE10	NM_199382.1	2,3-bisphosphoglycerate mutase	Erythrocyte development, glycolysis
5VF5	XR_085832.2	rattus norvegicus ubiquitin carboxyl-terminal hydrolase CYLD-like	Ubl conjugation pathway, Wnt signaling pathway
5VF7	NM_001047862.1	NADH dehydrogenase (ubiquinone) 1 alpha subcomplex, 8 (Ndufa8)	Unknown
5VF8	NM_001014762.1	p53 and DNA damage regulated 1 (Pdrg1)	Protein folding
5VG3	NM_001173380.1	mannosidase, beta A, lysosomal-like (Manbal)	Unknown
5VG5	NM_017326.2	calmodulin 2 (Calm2)	Activation of adenylate cyclase activity, Calcium-mediated signaling, Positive regulation of nitric-oxide synthase activity, Regulation of high voltage-gated calcium channel activity, Ryanodine-sensitive calcium-release channel activity, Store-operated calcium channel activity, Response to amphetamine and corticosterone stimulus
5VG7	NM_001004283.1	eukaryotic translation initiation factor 3, subunit D (Eif3d)	Protein biosynthesis
5VH3	XR_146921.1	uncharacterized LOC100911473	
5VH4	NM_053439.1	RAN, member RAS oncogene family (Ran)	Cell cycle, Cell division, Mitosis, Protein transport, Transport
5VH7	NM_001105717.2	dihydropyrimidinase-like 2 (Dpysl2)	Differentiation, Neurogenesis
5VH6	XM_001070261.3	cadherin-related family member 2 (Cdhr2)	Homophilic cell adhesion, Negative regulation of cell growth

a Accession number of the best match sequence.

b Identification based on sequence similarity.

c Biological process according to http://www.uniprot.org/doi:10.1371/journal.pone.0053605.t002.

**Table 3:** Identification of down-regulated cDNA sequences.

Clone	Accession N° of matching sequence^(a)^	Putative identification^(b)^	Biological process^(c)^
4NG6	NM_012629.1	prolactin (Prl)	STAT protein import into nucleus, Cellular response to hormone stimulus, Circadian rhythm, Lactation, Multicellular organismal response to stress, Ovulation cycle, Parturition, Positive regulation of JAK-STAT cascade, Epithelial cell proliferation, Regulation of ossification, Response to drug, estradiol stimulus, ethanol, lipopolysaccharide and nutrient
5ND3	XM_003751705.1	amphyphisin-like (Amph2)	Differentiation, Endocytosis
5ND7	NM_001034848.2	growth hormone 1 (Gh1)	Cellular response to insulin stimulus, Female pregnancy, Lung alveolus development, Negative regulation of neuron death, Neuroblast proliferation, Positive regulation of growth, Multicellular organism growth, Neurogenesis, Steroid hormone biosynthetic process, Response to cytokine stimulus, Light stimulus, Peptide hormone stimulus and signal transduction
7NB6	NM_017166.1	stathmin 1 (Stmn1)	Differentiation, Neurogenesis
9ND5	NM_017088.2	GDP dissociation inhibitor 1 (Gdi1)	Negative regulation of axonogenesis, Positive regulation of GTPase activity, Protein transport, Response to calcium ion
11ND5	NM_001109620.1	mitochondrial ribosomal protein L10 (Mrpl10)	Ribosome biogenesis, Translation

a Accession number of the best match sequence.

b Identification based on sequence similarity.

c Biological process according to http://www.uniprot.org/doi:10.1371/journal.pone.0053605.t002.

**Table 4:** Expression levels of each gene considered obtained by 2-ΔΔC_T_ method in the different groups of rats.

		Napa	Pfn2	Casp3	Pdgr1	Ywhz	Stmn1	Bpgm	Actr3	Pea15	Tiprl	Cplx1	Trim32	Ran	Amph2	Tomm 20	Nrd1
naïve n = 5	1.00±0.09		1.00±0.39	1.00±0.08	1.00±0.16	1.00±0.39	1.00±0.29	1.00±0.21	1.00±0.18	1.00±0.54	1.00±0.45	1.00±0.21	1.00±0.19	1.00±0.31	1.00±0.08	1.00±0.25	1.00±0.25
CFC n = 5	1.86±0.14***		2.64±0.14***	3.14±0.09***	2.23±0.23***	3.25±0.18***	2.02±0.24**	2.07±0.34**	1,64±0.12	1.15±0.09	1.570±0.240	1.36±0.11	1.40±0.07	1.44±0.27	0.43±0.08***	1.90±0.60**	1.470±0.09*
SO n = 5	1.12±0.17		0.94±0.25	1.03±0.20	0.87±0.13	0.93±0.20	1.16±0.25	0.95±0.38	2.25±0.91***	1.51±0.32*	2.54±0.68***	1.27±0.24	1.30±0.35	1.02±0.14	0.75±0.17	1.30±0.342	1.08±0.18
explor n = 5	1.05±0.18		1.47±0.59	1.22±0.56	1.31±0.58	1.02±0.14	1.04±0.57	1,21±0,44	0.79±0.21	1.03±0.28	0.83±0.22	1.73±0.27***	1.59±0.29**	2.04±0.88**	1.12±0.20	2.02±0.37**	1.39±0.29*

*indicates p<0.05.

**indicate p<0.01.

***indicate p<0.001.

### CFC induces changes in protein synthesis

**Figure 4:**
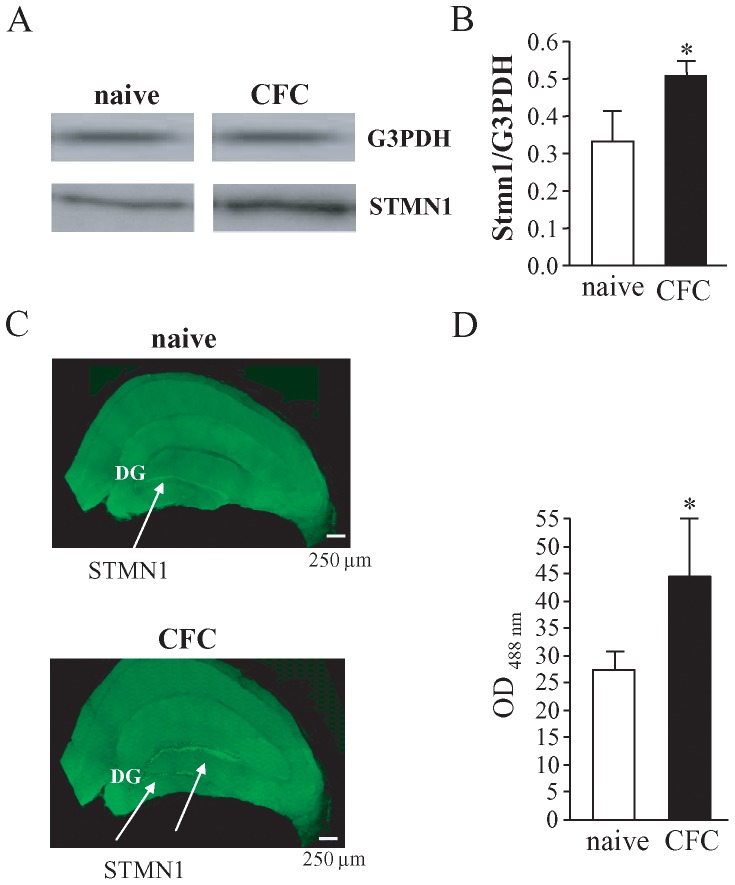
Two days after conditioning, STMN1 was highly expressed in the brain of CFC rats with respect to naïve rats. (A) Western blotting performed with brain samples from naïve and CFC rats shows that the blot obtained from brain samples of naïve rats was more weakly positive for STMN1 compared to the one obtained from brain samples of CFC rats, whereas the G3PDH expression was comparable in naïve and CFC animals (upper blots). The blots are representative of the result obtained from three independent experiments. (B) Histograms showing the ratio of the optical density between STMN1 and G3PDH calculated for naïve and CFC animals. * indicates p<0.01, Unpaired *t* test. (C) Dentate gyrus (DG) of rats subjected to CFC had a higher level of STMN1. It is localized in DG of rats subjected to CFC and sacrificed 2 days later without receiving the stimuli of the training sessions again. The results shown are representative of all the brains from three independent experiments. (D) Histograms showing the ratio of the optical density between G3PDH and STMN1 calculated for naïve and CFC animals. * indicates p<0.01.

To test whether changes in gene expression were accompanied by changes in protein synthesis, for example, we evaluated whether an increase of the STMN1 synthesis occurred during the consolidation phase of CFC. The blots in Figure 4A show an increase of the synthesis of STMN1 in the mid-temporal areas of brains dissected from rats subjected to CFC and sacrificed 2 days later compared to the same brain regions in naïve rats, which never entered the conditioning apparatus. No changes in the protein synthesis were detected with G3PDH used as an internal control. The ratio of the optical density between G3PDH and STMN1 was calculated and in CFC rats were significantly higher than in naïve rats (naïve: 0.207±0.016, n = 5; CFC: 0.392±0.054, n = 5; Unpaired t test, t = 3.285, df  = 8, p = 0.0047) (Fig. 4B). This result confirms that the over-expression of the gene coding for STMN1 observed by qRT-PCR leads to an increase of the protein synthesis.

STMN1 is known to be highly expressed in the lateral amygdala and in associated thalamic and cortical structures [28], [29]. Our data show that 2 days after CFC STMN1 was also highly expressed in the dentate gyrus of the hippocampus. As shown in Figure 4C, a faint immunoreactivity of STMN1 by immunohistochemical staining was observed in the dentate gyrus (DG) of naïve rats (27.30±1.75, n = 5), while a strong one was observed in the DG of CFC rats sacrificed 2 days after CFC (44.44±3.40, n = 5; Unpaired t test, t = 4.482, df  = 8, p = 0.002). No immunoreactivity was observed when the primary antibody was replaced with PB (data not shown).

## Discussion

Emotional memory tends to be long-lasting and plays an important role in regulating behavioural responses in animals. Because it is essential for survival, the memory of fear is easily established and very resistant to extinction. In the CFC paradigm, an emotionally neutral CS is paired with an aversive unconditioned stimulus, (US, foot-shocks), during the acquisition phase. The CS elicits a conditioned fear response during expression phase, without US presentation, because it acquires aversive reinforcing properties [20], [21]. After a single training, animals easily learn and retain for a long time the association between new surroundings (CS) and a given number of aversive stimuli (US) which are administered to them during their exploratory activity in the conditioning apparatus.

Many aspects of learning and memory storage in the mammalian brain involve cellular and molecular mechanisms that are driven by gene expression that is neural circuitries specific [30], [31]. Within the mammalian brain, one of the best understood memory-related neural circuitries is that which controls fear conditioning. In CFC the association between a context and aversive stimuli occurs so that this form of associative learning is very useful for studying the mechanisms underlying the consolidation of fear and spatial memory.

Rats trained in CFC and tested 2 days later (Fig. 1), during retrieval testing showed freezing for at least 65% of the time, whereas rats which had freely explored the experimental apparatus (exploration) and rats which received the same number of aversive shocks in the same apparatus, but temporally compressed (shock-only) did not exhibit freezing, indicating that our CFC training protocol produced robust and durable levels of conditioned fear.

Accumulating evidence indicates that contextual fear learning and memory processing is mediated by neural circuits comprising reciprocally connected brain regions, and the transformation of initially vulnerable traces into resistant ones involves the coordinated activation of the hippocampus [32] and the amygdala [33] and distributed cortical areas including retrosplenial cortices. Therefore, long-term memory seems to depend on the feedback between these areas rather than on any single structure. Several models have proposed that the hippocampus plays a pivotal role in organizing remote memory storage that would consist in actively modifying connectivity in distributed cortical networks [34]. This role suggests that changes in the morphology of hippocampal or cortical neurons should take place during the formation of recent or remote memories.

Moreover gene expression is subject to a host of regulatory mechanisms, expanding the way in which cells can regulate their protein composition and setting the bases for the complexity of the morphology and connectivity of neuronal cells. For these reasons, as a first step in a more exhaustive investigation, we analyzed the gene expression in the medial-temporal regions including the hippocampus and amygdala 2 days after the acquisition of a CFC paradigm.

The screening of SSH-cDNAs libraries obtained from mid-temporal brain areas of rats sacrificed 2 days after CFC and naïve rats (i.e rats which never entered the conditioning apparatus) showed, in both the forward and the reverse libraries, a pool of genes candidates to be differentially expressed.

The analysis of the expression of some randomly selected clones has shown that CFC modulates the expression of genes coding for proteins involved in signal transduction (YwhazM Pdrg1), synaptic activity (Napa, Amph2), synaptic remodelling (Pfn2, Casp3, Stmn1) and different cellular processes (Bpgm), whereas only electric shocks induce the modulation of the expression of genes (Actr3, Pea15, Tipr1) coding for proteins involved in the signal transduction. The exploration of the context modulates the expression of genes (Cplx1, Trim32, Ran) coding for proteins involved in the synaptic activity, protein turnover and other functions respectively. In both CFC and exploration rats the expression of the genes Tomm20 and Nrd1 were higher than in naïve or SO rats. These results clearly indicate that the association of context and aversive stimuli (electric shocks) determines a modulation in the expression of specific genes.

Our data show that the gene coding for NAPA was over-expressed 2 days after CFC. This gene encodes a member of the soluble NSF attachment protein (SNAP) family. SNAP proteins play a critical role in the docking and fusion of vesicles to target membranes as part of the 20S NSF-SNAP-SNARE complex. The encoded protein plays a role in the completion of membrane fusion by mediating the interaction of N-ethylmaleimide-sensitive factor (NSF) with the vesicle-associated and membrane-associated SNAP receptor (SNARE) complex, and stimulating the ATPase activity of NSF [36]. The increased expression after CFC suggests that the consolidation phase of memory needs a constant and conspicuous release of neurotransmitter.

The intense pre-synaptic activity after CFC also seems to be sustained by an increase in the expression of the genes coding for PFN2, which is a prominent regulator of actin dynamics [37], [38] and YWHAZ, a protein involved in the regulation of actin cytoskeleton [39]. Though genetic, physiological and biochemical studies have led to controversial interpretations of the role of PFN2 in synaptic architecture and function, it is known that PFN2 is associated with effectors of exocytotic and endocytotic pathways [40] and over-expressed PFN2 was observed to translocate into dendritic spines of cultured neurons in an activity-dependent manner [41]. Moreover, fear conditioning correlated with profilin enrichment in dendritic spines of the rat amygdala [42].

Recently, in hippocampal slices it has been found that the Ywhaz gene is up-regulated after repetitive glutamate stimulation which mimicks a long-lasting L-LTP and our results show an up-regulation of this gene specifically after CFC, suggesting an involvement of the YWHAZ/14,3,3 ζ protein in neuronal plasticity. These results are consistent with several studies which point to an important role of 14-3-3 proteins in the nervous system. Genetic knock-out of 14-3-3 in *Drosophila* revealed an impairment of learning and synaptic plasticity [43]. In support of a similar function in mammals, it has been shown that 14-3-3 proteins are required for a pre-synaptic form of long-term potentiation in the mouse cerebellum [44].

Caspases are cysteine proteases with well-established functions in the execution of apoptosis. Interestingly, active caspases have been also detected in non-apoptotic cells. In neurons, mitochondria and caspases are present in dendrites, axons and pre- and post-synaptic terminals, and there is evidence that caspases can be activated in dendrites, synaptosomes and growth cones [45]. It has been reported that caspases play non-apoptotic roles in the structural remodelling of hippocampal neuron synapses [45] and in bird song learning [46]. Recently, it has been shown that caspases including CASP3 are involved in the induction of synaptic depression in rat hippocampus [47], [48]. CASP3 can be activated by NMDA stimulation without inducing cell death. The over-expression of the gene coding for CASP3 we observed 2 days after CFC might sustain non apoptotic phenomena such as those described above.

Two days after CFC, we also detected an over-expression of the Pdgr1 gene coding for P53 and DNA-damage regulated 1 protein which is a nuclear phosphoprotein up to date known to bind DNA to activate mechanisms of DNA repair, cell survival and axonal outgrowth [49]. In addition, our data can account for a role of PDRG1 in cognitive processes.

We have focused on the modulation of the gene coding for STMN1, a regulator of microtubule formation. Our results show that CFC induces an increase of the expression of the protein 2 days after conditioning. Up to now STMN1 has been identified as being crucially involved in fear processing in rodents [35] and also in humans [50], [51]. Our data also ascribe to STMN1 a role in the consolidation of a form of associative learning in which the context and aversive stimuli are associated. This form of learning involves both the amygdala and the hippocampus. STMN is known to be highly expressed in the lateral amygdala and in associated thalamic and cortical structures [28], [29], whereas our data show that 2 days after CFC STMN1 is also highly expressed in the DG of the hippocampus. The hippocampus is believed to play a role in processing information relative to the context in which emotionally salient experiences occur. We observed a faint immunoreactivity of STMN1 by immunohistochemical staining in the DG of naïve rats while a strong one was observed in the DG of CFC rats sacrificed 2 days after conditioning

This result is not surprising because the DG is the primary relay station for incoming inputs to the hippocampus and the integrity of the DG is essential for establishing a coherent representation of the context to which emotional experiences, either hedonistic or aversive, are bound [52]–[54]. Moreover, in the DG adult neurogenesis can occur and, starting from 1991 [55], STMN1 has been detected in germinal brain areas with neurogenic potential including the DG which is classically thought to be necessary for the acquisition and expression of associations between contexts and internal states. Our data show that the DG also plays a role in the consolidation of spatial memory related to an emotional state. The animals we used did not receive the stimuli of the training sessions (context and electrical shocks) again so that the biological changes we observed can be related only to the consolidation and/or to the storage of engrams.

In our screening, only the Amph2 gene was down-regulated 2 days after CFC. This gene encodes several isoforms of a nucleocytoplasmic adaptor protein, one of which was initially identified as a MYC-interacting protein with features of a tumor suppressor. Data collected up to now indicate that the isoforms that are expressed in the central nervous system (BIN1/AMPH2 proteins) may be involved in synaptic vesicle endocytosis and may interact with dynamin, synaptojanin, endophilin, and clathrin for the formation and/or maintenance of endocytic recycling compartment derived tubules [56], [57]. The reduced expression of this gene we detected suggests a complex regulation of the pre-synaptic machinery during the consolidation of CFC which is likely time-dependent. Further investigations are necessary to better clarify this issue.

A group of genes whose expression was modulated by CFC includes genes that code for proteins whose functions are currently not clarified in detail and therefore it is difficult to assign functional significance to the observed changes in expression. They are the genes coding for BPGM, a trifunctional enzyme with the function of synthase and phosphorylase mutase and for NRD1 a metal-protease with proteolitic function.

Overall, our results show how in CFC a modulation in expression of a wide range of genes occurs. These genes code for proteins involved in multiple functions, some of which currently cannot be clearly framed in the mechanisms that are classically recognized as the basis of the induction and retention of learning and memory. Therefore, it would be interesting to complete the analysis of the expression of all genes screened in our SSH-cDNAs libraries in further investigations in order to better understand the molecular and cellular mechanisms related to the consolidation of the association between context and emotional state. With this aim, it could also be interesting to do a time course of the gene expression performing the analysis even at other times after CFC.

## Supporting Information

Figure S1 **Regression lines for three genes showing predicted regression lines and actual means.** The most stable and consistent control genes would have the lowest slope and closest fit to the regression line. *G3PDH* (first from bottom) had the highest and *RPL13A* (second from bottom) the second highest stability indices. *Actin* (third from bottom) had the lowest stability index.
(PPT)

Table S1 Means ± SEM of CT value for the three housekeeping genes tested in the samples used.
(PPT)
