# Supplementary figures and images for: Modulation of Gene Expression in Contextual Fear Conditioning in the Rat
Source: PLoS One. 2013 Nov 21;8(11):e80037. doi: 10.1371/journal.pone.0080037 (PMC3837011; doi:10.1371/journal.pone.0080037)

## Slide 1
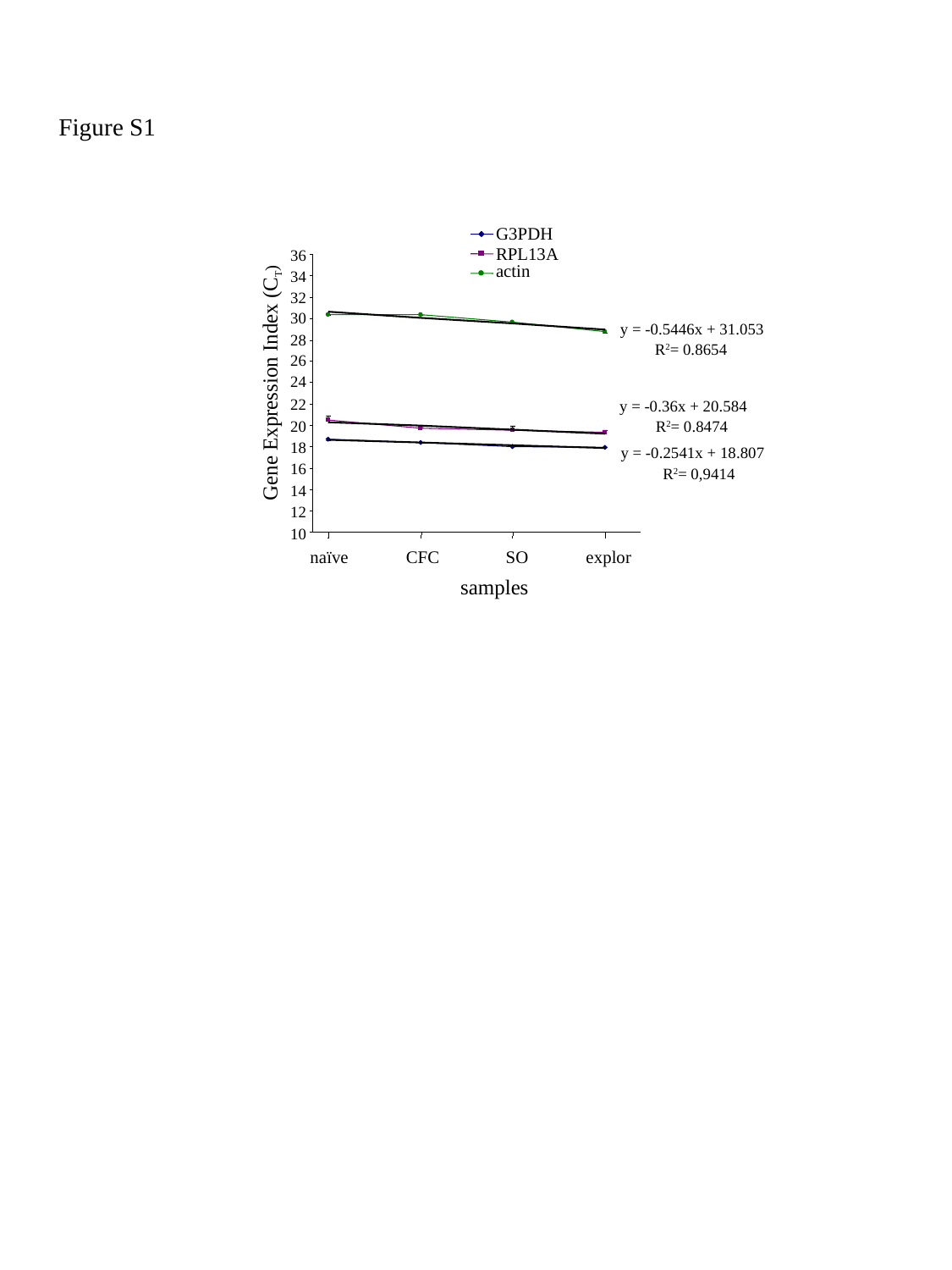

Figure S1
G3PDH
RPL13A
36
actin
34
32
30
y = -0.5446x + 31.053
28
Gene Expression Index (CT)
 R2= 0.8654
26
24
22
y = -0.36x + 20.584
20
 R2= 0.8474
18
y = -0.2541x + 18.807
16
 R2= 0,9414
14
12
10
naïve CFC SO explor
samples

Supplement: Figure S1 — Regression lines for three genes showing predicted regression lines and actual means. The most stable and consistent control genes would have the lowest slope and closest fit to the regression line. G3PDH (first from bottom) had the highest and RPL13A (second from bottom) the second highest stability indices. Actin (third from bottom) had the lowest stability index. (PPT) [file pone.0080037.s001.ppt]
