# Supplementary material for: Modulation of Gene Expression in Contextual Fear Conditioning in the Rat
Source: PLoS One. 2013 Nov 21;8(11):e80037. doi: 10.1371/journal.pone.0080037 (PMC3837011; doi:10.1371/journal.pone.0080037)
Supplement: Table S1 — Means ± SEM of CT value for the three housekeeping genes tested in the samples used. (PPT) [file pone.0080037.s002.ppt]

## Slide 1
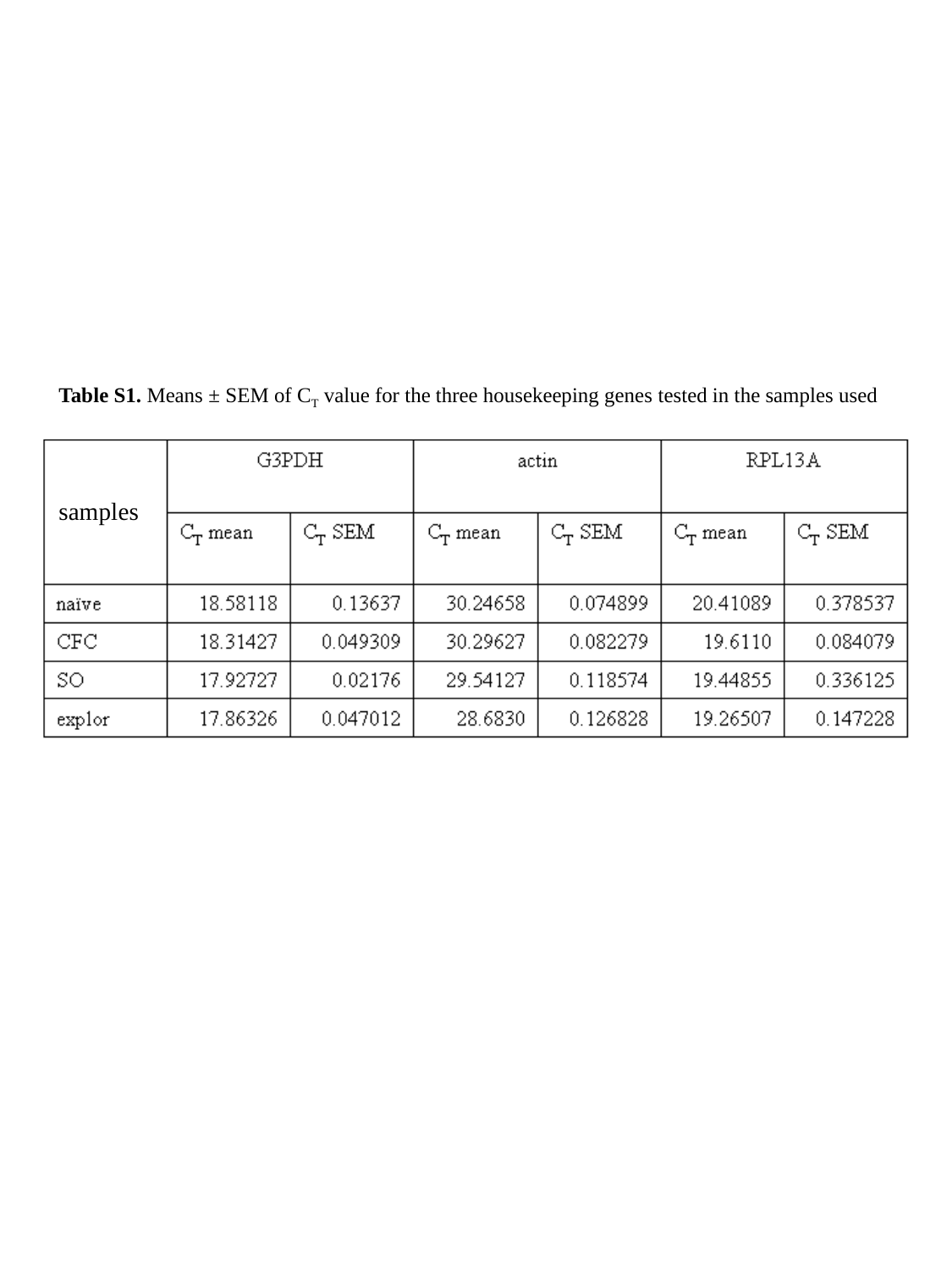

Table S1. Means ± SEM of CT value for the three housekeeping genes tested in the samples used
samples
